# Histopathology and treatment of a huge overhanging filtering bleb

**DOI:** 10.1186/s12886-016-0353-7

**Published:** 2016-10-06

**Authors:** Ping-bo Ou-yang, Xin Qi, Xuan-chu Duan

**Affiliations:** 1 Department of Ophthalmology, Second Xiangya Hospital, Central South University, Changsha, Hunan Province 410011 People’s Republic of China; 2 Institution of Ophthalmic Center, Second Xiangya Hospital, Central South University, Changsha, Hunan Province 410011 People’s Republic of China; 3 Second Xiangya Hospital, Central South University, No.139, Remmin Middle Road, 410011 Changsha, Hunan Province People’s Republic of China

**Keywords:** Overhanging filtering bleb, Histopathology, Immunohistochemistry

## Abstract

**Background:**

The giant filtering bleb encroaching onto the corneal surface is a rare occurrence in our and other’s clinical experience (Kapoor and Syed, Int. Ophthalmol 31(5):403–404, 2011), even in patients having had a trabeculectomy with mitomycin C, and how it developed is debated. In this paper, we report a patient who developed a huge overhanging filtering bleb after trabeculectomy, and present our intraoperative photographs, histopathology and immunohistochemistry results.

**Case presentation:**

A 62-year-old female visited our hospital due to the giant filtering bleb encroaching onto the corneal surface which was about 6 mm × 8 mm × 3 mm. We dissected the filtering bleb from the cornea and present the histopathology and immunohistochemistry results of it.

**Conclusion:**

The results from histopathology and immunohistochemistry in this study are consistent with the filtering cicatrix hypothesis. However, our finding that the overhanging blebs had tight connections with the corneal tissue or corneoscleral limbus, rather than simply leaning on it, might be highly related to their development and still needs to be further studied.

**Electronic supplementary material:**

The online version of this article (doi:10.1186/s12886-016-0353-7) contains supplementary material, which is available to authorized users.

## Background

An overhanging filtering bleb is a uncommon postoperative complication of trabeculectomy [1], and is thought to be increasing with the greater use of antimetabolites in glaucoma filtering surgery [2]. The term overhanging filtering blebs refers to oversized filtering blebs that project over the cornea [2–4]. Patients commonly suffer symptoms that increase with the growth of the bleb. Symptoms are constant foreign body sensation, excessive tearing, sensitivity to light and visual disturbances, and are considered to be caused by tear film instability, increased astigmatism and occlusion of the visual axis by the large blebs [2–4]. Patients also generally complain of poor cosmesis [4]. Excision of overhanging filtering blebs is recommended for patients with seriously compromised comfort and visual function [1, 2, 5]. However, how it developed is debated. In this paper, we report a patient who developed a huge overhanging filtering bleb after trabeculectomy, and present our intraoperative photographs, histopathology and immunohistochemistry results, and discussed about the mechanism of overhanging blebs formation.

## Case presentation

**Fig. 1:**
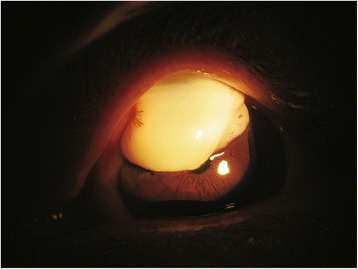
Slit-lamp examination of the overhanging filtering bleb. The giant filtering bleb was about 6 mm × 8 mm × 3 mm. This cystic and thin-walled bleb was not movable and encroached onto the superior part of the cornea, shading the pupil. The bleb was avascular, although its edge showed neovascularization

**Fig. 2:**
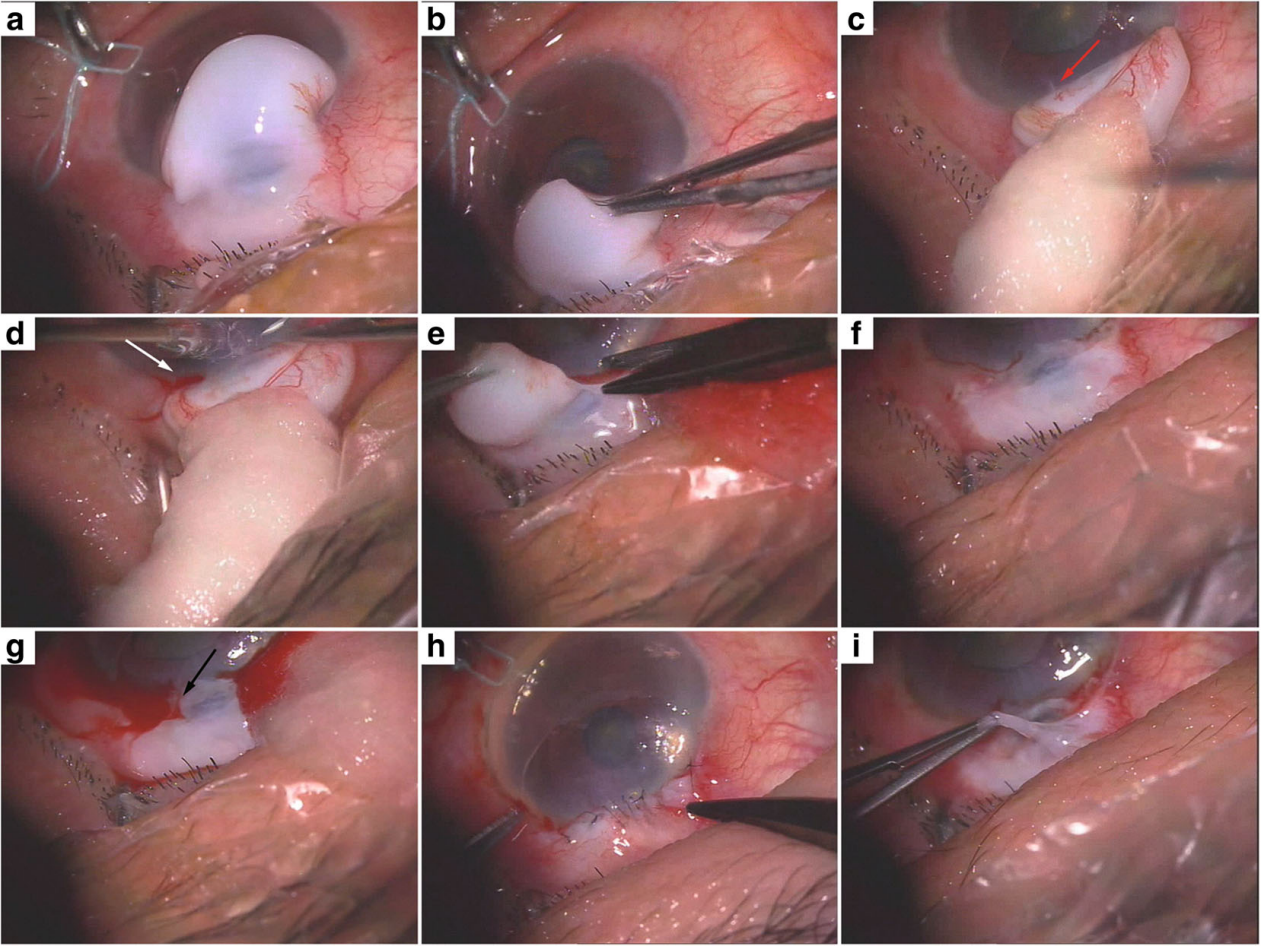
Intraoperative photographs of the resection operation of the overhanging filtering bleb. **a** The filtering bleb was sufficiently thin-walled that the scleral incision could be seen through the bleb. **b** The overhanging portion of the bleb was bluntly dissected from the cornea gently, without bleeding. **c** The complete marginal vascular arcade at the backside of the bleb, fibrous bundles at the limbus (*red arrow*), and epithelium defect of the corresponding part of cornea. **d** Bleeding during blunt dissection of the fibrous bundles (*white arrow*). **e**, **f** The overhanging portion of the bleb was lifted with forceps and excised with scissors just anterior to the limbus. **g** An aqueous humor leak was marked by blood (*black arrow*) and followed by the shallowing of the anterior chamber. **h** The incision was continuously sutured with 10-0 nylon wire with an anterior bite through the cornea, just anterior to the limbus, and a posterior bite through the conjunctiva and Tenon’s tissue posterior to the bleb. **i** The conjunctiva was fragile and tore during suturing although lifted gently with smooth forceps

A 62-year-old female visited our hospital due to foreign body sensation and visual disturbances in her left eye that had been present for 2 years without an obvious cause. Three years before, the patient underwent trabeculectomy with mitomycin C (MMC) application (0.25 mg/ml for 3 min). Intraocular pressure (IOP) had been well-controlled without any postoperative anti-glaucoma drugs. Best-corrected visual acuity was 20/1000 in this eye. A large filtering bleb was identified upon slit-lamp examination, which prevented measuring IOP by Goldmann applanation tonometry. The giant filtering bleb was unmovable and encroached onto the corneal surface, shading the pupil. Its length was about 6 mm, the maximum width of the corneal component was about 8 mm, and the maximum altitude was 3 mm (Fig. 1). Additionally, the filtering bleb was avascular and thin-walled. The scleral incision could be seen through the bleb (Fig. 2a).

**Fig. 3:**
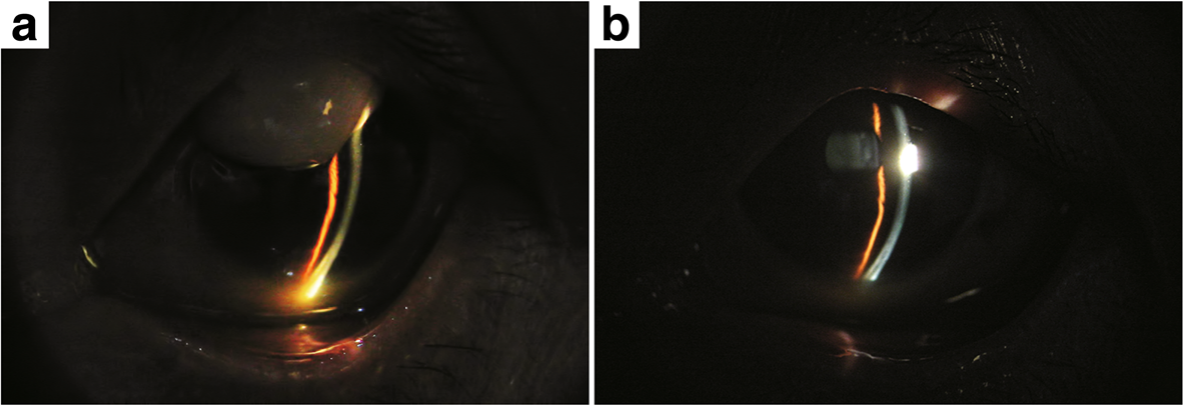
The anterior chamber deepened after the resection operation of the overhanging filtering bleb. **a** Preoperation. **b** Postoperation

**Fig. 4:**
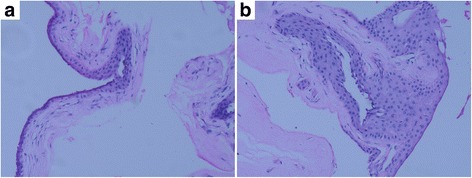
Histopathology of the overhanging filtering bleb: Both of figure **a** and **b** indicate neoplasm-like hyperplasia covered with atrophic squamous epithelium and filled with mucus-like loose connective tissue and several interspersed star-shaped fibroblast cells. There was no evidence of inflammation or tumors

**Fig. 5:**
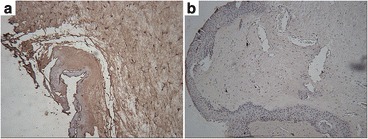
Immunohistochemistry of the overhanging filtering bleb for (**a**) TGF-β_2_, strongly positive and (**b**) COL1A1, negative

The patient underwent resection of the overhanging filtering bleb, which was bluntly dissected from the cornea, and no bleeding occurred (Fig. 2b). After dissection, we could see the complete marginal vascular arcade at the backside of the bleb, fibrous bundles at the limbus, and the epithelial defect in the corresponding part of cornea (Fig. 2c). The fibrous bundles were also dissected bluntly, after which bleeding occurred (Fig. 2d). The overhanging portion of the bleb was then excised just anterior to the limbus (Fig. 2e, f). An aqueous humor leak was marked by blood and followed by the shallowing of the anterior chamber (Fig. 2g). The incision was continuously sutured with 10–0 nylon wire (Fig. 2h). The conjunctiva was particularly fragile, tearing during suturing even though it was lifted gently with smooth forceps (Fig. 2i). The anterior chamber was formed by air injection just prior to the end of the operation. There was no leakage around the margin of the excision the day after operation, the patient’s visual acuity kept 20/200 and showed good IOP (15–18 mmHg) during the first week after the operation (Additional file 1), and with the air filled in, the anterior chamber deepened than it used to be. Three months after surgery, the patient showed good visual acuity (20/100) and IOP (15 mmHg) during the follow-up. Notably, the anterior chamber deepened postoperatively even though the air was completely absorbed (Fig. 3). Histopathology of the excised corneal overhanging filtering bleb showed polypoid structures covered with flattened corneal epithelium and filled with myxedematoid loose connective tissue. This connective tissue had a small number of stellate sporadic fibroblasts, and its diagnosis was neoplasm-like hyperplasia (Fig. 4). The tissue was strongly positive for transforming growth factor-beta 2 (TGF-β_2_; Fig. 5a) and negative for Collagen α1 Type I (COL1A1; Fig. 5b), detected by immunohistochemistry.

## Conclusions

Overhanging blebs can occur days [6] to years [7] after the procedure in patients from 12 to 82 years old [4, 6] and their incidence appears to be increasing with the current liberal use of antimetabolites in glaucoma filtering surgery [2, 7]. The giant filtering bleb encroaching onto the corneal surface of this patient was about 6 mm × 8 mm × 3 mm, which is a uncommon occurrence in our and other’s clinical experience [8, 9]. In our patient, the conjunctiva of the bleb was fragile (Fig. 2i), which may have been the result of MMC application during her trabeculectomy.

How overhanging blebs develop is debated. Sheie et al. [1] hypothesize that an overhanging bleb is a filtering cicatrix that has been massaged downward over the cornea by the action of the eyelid, and that the bleb is in contact only with the cornea surface. To test whether scarring played a role in our patient, we performed immunohistochemistry for TGF-β_2_, which is thought to function in the scarring of filtering blebs. Indeed, the tissue was strongly positive for this protein (Fig. 5a). Further, our sample was negative for COL1A1, which is thought to be an anti-scarring protein (Fig. 5b). These results are consistent with Sheie and colleagues’ filtering cicatrix hypothesis. The diagnosis of our patient was neoplasm-liking hyperplasia (Fig. 4), which may also support this model. However, the view of Sheie and colleagues that overhanging blebs contact only the corneal surface was not consistent with our observations. During the operation, bleeding occurred during blunt dissection of the fibrous bundles (Fig. 2d). Such a bleeding suggests that the overhanging filtering bleb had tight connections with the corneal tissue or corneoscleral limbus, rather than simply leaning on it.

**Fig. 6:**
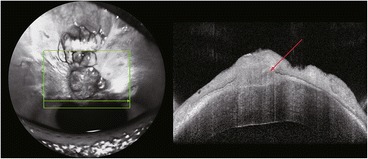
OCT image of the anterior segment of a small overhanging filtering bleb from another patient. The *red arrow* indicates the little tissue space between the cornea or corneoscleral limbus. The *green arrow* indicates the OCT scaling line

A competing hypothesis by Ulrich and colleagues [10] postulates that the formation of overhanging blebs involves aqueous humor dissection between the corneal epithelium and stroma. The internal structure of blebs can be revealed by Ultrasound Biomicroscopy (UBM) or Optical Coherence Tomography (OCT). Ito et al. [3] have reported an overhanging bleb that had developed on top of a primary bleb. The two blebs were separated by a clear membrane border. The inner structure of both blebs had a water cleft and a low-density area. Kim WK et al. [5] have demonstrated via OCT that an overhanging filtering bleb was multiloculated cystic, which was confirmed by histopathology. The biopsy specimen of the overhanging filtering bleb reported by Grostern RJ et al. [11] was also multiloculated cystic. We have examined another patient’s small overhanging filtering bleb with OCT, which revealed that there was little tissue space between the cornea or corneoscleral limbus (Fig. 6). The histopathology findings of Kim and colleagues [5]., Grostern and colleagues [11] and our results show that overhanging blebs are covered with flattened corneal epithelium, which would support Ulrich and colleagues’ hypothesis.

**Fig. 7:**
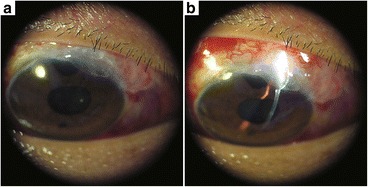
Slit-lamp examination of the overhanging filtering bleb from another patient. The bleb was flattened with pressure from cotton pillow (**a**) but refilled in several seconds in the absence of pressure (**b**). The time was too short for the slit-lamp light to be adapted properly in Picture a

Others [12] have proposed that gravity also takes part in the formation of overhanging filtering blebs. Since MMC has the function of anti-proliferation on fibroblast cells, and with the wash of aqueous, the filtering bleb will get thinner gradually, especially on the aged people. Meanwhile, the gravity keeps working on the overweight bleb, causing huge overhanging filtering bleb formed. In our patient, the temporary ocular hypotension which might be caused by excessive aqueous over filtration could provide permissive conditions for the action of gravity. The gravity kept working on the bleb’s tissue even the IOP back to normal, and gradually formed into the huge overhanging filtration bleb. The second overhanging filtering bleb we have observed was flattened after pressure was applied with a cotton pillow but filled in rapidly (several seconds) in the absence of pressure (Fig. 7). This may also reflect excessive aqueous over-filtration in an overhanging filtering bleb. However, Sony and colleagues [6] have reported a large diffuse overhanging bleb extending from the 8 o’clock to 10 o’clock position, which does not correlate with the action of the eyelid nor gravity.

In summary, the mechanism for overhanging blebs formation may be complex. The factors discussed here, such as scar hyperplasia, the action of gravity, the action of the eyelid and excessive aqueous over-filtration may interact and together contribute to the formation of overhanging filtering bleb. However, our finding that the overhanging blebs had tight connections with the corneal tissue or corneoscleral limbus, rather than simply leaning on it, might be highly related to its development and still needs to be further studied.

## Additional file

Additional file 1: Fundus photography. the fundus photography of the patient after the operation,the cup-disc ratio was around 0.6, and the fundus was clear, there was no sign of chorioretinal folds and disc oedema. (TIF 9217 kb)
