# Supplementary figures and images for: Histopathology and treatment of a huge overhanging filtering bleb
Source: BMC Ophthalmol. 2016 Oct 6;16:175. doi: 10.1186/s12886-016-0353-7 (PMC5053132; doi:10.1186/s12886-016-0353-7)

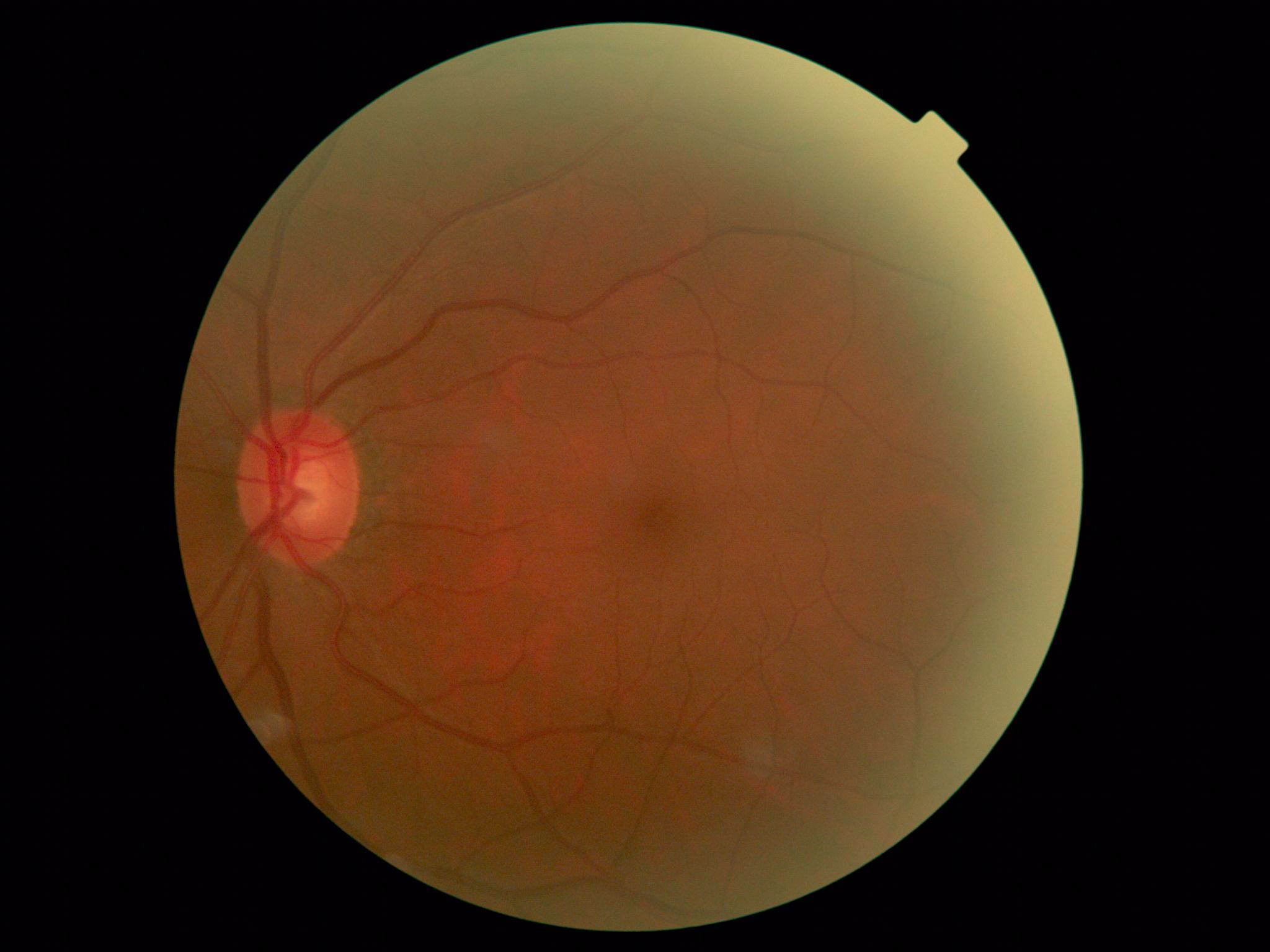

Supplement: Additional file 1: — Fundus photography. the fundus photography of the patient after the operation,the cup-disc ratio was around 0.6, and the fundus was clear, there was no sign of chorioretinal folds and disc oedema. (TIF 9217 kb) [file 12886_2016_353_MOESM1_ESM.tif]
